# Artificial Intelligence in cardiopulmonary resuscitation training – A scoping review

**DOI:** 10.1016/j.resplu.2025.101175

**Published:** 2025-11-22

**Authors:** Timo de Raad, Olfa Chakroun-Walha, Brenna Leslie, Robert Greif, Sabine Nabecker

**Affiliations:** a Department of Pediatric Intensive Care, University Medical Centre Utrecht, Utrecht, the Netherlands; b Emergency Department, Habib Bourguiba University Hospital, Faculty of Medicine, Sfax University, Tunisia; c Mount Sinai Hospital, Sinai Health, Toronto, Canada; d Faculty of Medicine, University of Bern, Switzerland; e Department of Anesthesiology and Pain Management, Sinai Health System, University of Toronto, Toronto, Canada

**Keywords:** Artificial intelligence, AI, Natural language processing, Cardiopulmonary resuscitation

## Abstract

**Objectives:**

This scoping review aimed to identify Artificial Intelligence methods used in cardiopulmonary resuscitation (CPR) training.

**Methods:**

Members of the writing group ‘Education for Resuscitation’ of the European Resuscitation Council 2025 guidelines used the PICOST format for this scoping review, which included only published randomized and non-randomized studies. Medline, Embase, Cochrane, Education Resources Information Center, Web of Science, and PubMed were searched from inception to July 2025. Title and abstract screening, full-text review, and data extraction were performed by two researchers in pairs. PRISMA reporting standards were followed. The review was registered at PROSPERO. Because the evidence was insufficient for a systematic review, we changed our initial plan and performed a scoping review.

**Results:**

The search identified 6977 citations. After removing 2521 duplicates, reviewing titles and abstracts yielded 43 articles for full-text review. Of these, 15 studies were included in the final analysis. Our findings reveal that Artificial Intelligence is being explored across key areas of CPR training, including its accuracy in detecting CPR quality parameters, providing real-time feedback, creating personalized training experiences, detecting and analyzing dialog segments during and after simulation, generating medical teaching illustrations, its capacity for interactive simulations, and answering laypersons’ medical questions.

**Conclusion:**

Artificial Intelligence shows potential for transforming CPR training via enhancing real-time feedback, enabling personalized learning, improving dialog analysis, facilitating content creation, and serving as an information source. The current evidence is dominated by proof-of-concept studies. Future research needs to establish the efficacy of Artificial Intelligence-supported CPR training compared to traditional methods.

## Introduction

Cardiac arrest presents a significant global health challenge, characterized by consistently low survival rates for both in-hospital and out-of-hospital cardiac arrests.^1^ In 2003, the International Liaison Committee on Resuscitation (ILCOR) Advisory Statement on Education and Resuscitation initially proposed a formula for survival in resuscitation.^2^ The formula included three interactive factors: guideline quality (science), efficient education of patient caregivers (education), and a well-functioning chain of survival at a local level (local implementation), all acting as crucial multiplicands in determining survival outcomes from resuscitation.^2^ It was later simplified to show ‘medical science × educational efficiency × local implementation = survival’.^3^ Consequently, current cardiopulmonary resuscitation (CPR) courses are designed to enhance lay providers' and healthcare professionals’ knowledge and skills in basic and advanced life support skills. This is achieved by adhering to current CPR guidelines, employing effective educational strategies, and tailoring the course content to the participants’ local circumstances.^4^, ^5^, ^6^, ^7^

Improving teaching and learning in CPR training is an evolving field. To successfully integrate new educational approaches or technologies, solid research is needed. Emerging tools powered by artificial intelligence (AI) hold great potential to transform CPR education for both laypeople and healthcare professionals. AI was explored in medical education for admissions, teaching, assessment, and clinical reasoning, but the recent Best Evidence in Medical Education (BEME) Guide No. 84 also concluded that there is an urgent need for ongoing research and ethical guidelines for the use of AI in medical education.^8^ While AI-driven tools may promise to enhance healthcare education, their current limitations should be acknowledged and addressed.^9^ By leveraging specific AI tools and techniques, such as natural language processing (NLP) and machine learning (ML), CPR education may be reimagined to deliver more personalized, adaptive, and effective learning experiences. Therefore, this scoping review aims to identify different AI methods and describe their use in CPR training.

## Methods

### Protocol

This review was conducted by members of the ‘Education for Resuscitation’ writing group of the European Resuscitation Council Guidelines 2025 (TDR, OCW, RG and SN). Initially, the intention was to perform a systematic review, but there was not enough evidence for such a review; therefore, the writing group decided to proceed with a scoping review instead. A review protocol was agreed upon *a priori* by the guideline writing group. This scoping review was conducted following a methodological framework for scoping reviews.^10^ We followed the Preferred Reporting Items for Systematic reviews and Meta-Analyses extension for Scoping Reviews (PRISMA-ScR) checklist^11^ and registered with the Prospective Registry for Systematic Reviews PROSPERO (CRD42024618790). The PRISMA checklist can be found in Appendix 1.

### Research question and eligibility criteria

The research question was structured using the ‘PICOST’ (Population, Intervention, Comparison, Outcome, Study Design, Timeframe) format:

***Population:*** Healthcare professionals (e.g., medical students, residents, nurses, paramedics) who are participating in CPR training of any level. Laypersons (e.g., general public, school staff, workplace employees) who are participating in CPR training or Basic Life Support (BLS) training.

***Intervention:*** Use of AI in CPR training (e.g. AI-driven chatbots for real-time feedback and guidance during simulation; machine learning algorithms for assessing key clinical skills like chest compression accuracy, rhythm detection, and airway management; NLP for automated debriefing and performance evaluation during team-based simulations; and AI-driven adaptive learning for personalized learning paths based on individual performance).

***Comparison:*** No such training or intervention, or traditional resuscitation education methods, such as lecture-based, low-fidelity simulations, and instructor-led feedback without AI integration.

***Outcomes:***

*Educational:* CPR skills performance in actual resuscitations and in educational settings (e.g., CPR quality, time to medication administration, initiation of CPR, time to defibrillation, chest compression fraction, etc.) (important outcome). Resuscitation knowledge (important outcome). Human factors (e.g., decision making, teamwork, team leadership, communication, workload, etc.) (important outcome).

*Clinical:* Good neurological outcome at hospital discharge/30-days (critical outcome), Survival at hospital discharge/30-days (critical outcome), Survival to hospital/event survival (critical outcome).

***Study design:*** Randomized controlled trials (RCT) and non-randomized studies (non-RCT, observational studies, interrupted time series, controlled before-and-after studies, cohort studies) were eligible for inclusion. In this review, we included studies related to immersive technologies, such as augmented reality and virtual reality, only if additional AI was used.

Unpublished studies (e.g., conference abstracts, trial protocols), editorials, commentaries, case studies, or case reports were excluded. Systematic reviews were only used to examine included studies. Studies were excluded if AI was used for purposes other than to enhance CPR training, e.g., a study linking personality traits to performance without an educational intervention.^12^ Similarly, a study exploring healthcare professionals attitudes toward technology and their effect on performance was excluded.^13^ We did not perform a Gray literature search. All relevant publications in any language were included as long as an English abstract was available.

***Time frame:*** from inception to 2 July 2025.

### Definitions

AI is the ability of machines to imitate human cognitive tasks such as image recognition, speech recognition, language processing, and content generation. AI models analyze large quantities of data to identify patterns and make highly accurate predictions or decisions across diverse tasks. AI tries to mimic how the human brain processes information by leveraging techniques like deep learning and neural networks. AI applications include generating text, recognizing images, and providing conversational responses, making it a powerful tool for solving complex problems and augmenting human capabilities.^14^

### Information sources and search strategy

The search strategy was developed in collaboration with an information specialist (BL). The searches included controlled vocabulary and keywords related to “resuscitation” OR “heart arrest” AND “artificial intelligence” OR “virtual reality” OR “augmented reality” AND “teaching” OR “learning” OR “education”. Although AI, augmented reality, and virtual reality are not synonymous, all three terms were included due to their overlap. The search was conducted across six electronic databases: Medline, Embase, PubMed (non-Medline records only), Web of Science, Education Resources Information Center (ERIC), and Cochrane Library. The search results were not restricted by age, language, year, geography, or species as long as an English abstract was available. The search results were managed using EndNote (Version 20.6, Clarivate, Philadelphia, USA) prior to being uploaded to Covidence (Veritas Health Innovation, Melbourne, Australia) for screening. Searches were performed from inception to December 15, 2024, and updated to July 2, 2025. Reference lists of included studies and reviews were searched to identify additional relevant articles. A hand-search of review articles and bibliographies did not reveal additional citations. The full search strategy is available in Appendix 2.

### Data extraction

The data-charting form was derived using Word (Microsoft, Redmond, Washington, USA) by TDR and SN. Both independently charted the data, discussed the results and in case of no agreement another researcher (RG) functioned as referee. Data included: author, year, country, aim, setting, study design, description of AI used, main findings, and limitations or comments. We listed all variables for which data were sought, and no assumptions or simplifications were made.

### Reporting method

Data from the included studies were charted using the pre-defined data extraction form described above. The data are summarized in a narrative form in the Results section, with details of the included publications presented in Table 2 and in Appendix 3.

### Changes to the original PROSPERO registration

After reviewing the available literature, the research group found that the evidence was very heterogenous and varied widely in design, purpose and outcome measures. The retrieved evidence primarily focused on the feasibility of AI in CPR training, we even could not follow the Synthesis Without Meta-analysis (SWiM) in Systematic Reviews Reporting Guideline.^15^ All that brought us to the decision to change for a scoping review to summarize better the found evidence. As our initial PICOST was very wide, it served also such a wider approach usually used for scoping reviews. Clinical outcomes and the clinical setting were included in the PICOST, but no results were found for these areas. The initial PROSPERO registration was not updated, but a note was left upon completion that the review was completed as a scoping review.

## Results

### Study characteristics

**Fig. 1:**
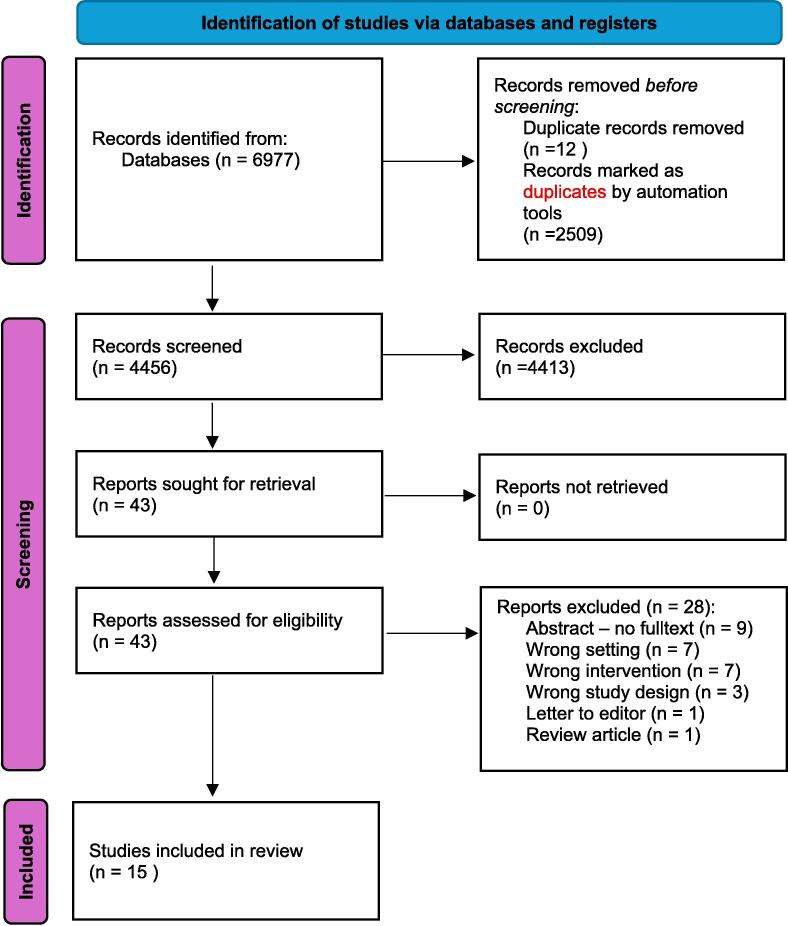
**Consort flow diagram**.

A total of 6977 studies were found; we identified 2509 duplicates using Covidence, and 12 duplicates were identified manually. Three reviewers (TDR, OC, and SN) screened 4456 titles and abstracts in pairs, excluding papers that did not meet the eligibility criteria. Disagreements were resolved through discussion or with the involvement of another team member (RG). The full text of the remaining 43 papers was then independently reviewed for eligibility by two reviewers (TDR, SN). Any remaining disagreements were discussed with a third team member (RG), leading to a final consensus on the selection of 15 included articles. Fig. 1 shows the PRISMA flowchart for this review. Since the decision was made to proceed as a scoping review, no risk of bias assessment or certainty of evidence process was applied.

Fifteen publications from Europe,^16^, ^17^, ^18^, ^19^, ^20^, ^21^, ^22^ Asia,^23^, ^24^, ^25^, ^26^ and North America^27^, ^28^, ^29^, ^30^ were included, none from low-resource settings. All studies were published between 2019 and 2025. All studies except one were proof-of concept studies outside of actual training situations,^16^, ^17^, ^18^, ^19^, ^20^, ^22^, ^23^, ^24^, ^25^, ^26^, ^27^, ^28^, ^29^, ^30^ only one randomized controlled study compared AI intervention with a traditional educational method.^21^ AI applications varied across studies, and the distribution of the different AI applications in CPR training is shown in Table 1. Table 2 summarizes the characteristics of the included studies and the respective outcomes regarding CPR training. Appendix 3 includes a more detailed account of the studies included.

**Table 1:** Artificial intelligence in cardiopulmonary resuscitation training – a scoping review. Overview of study characteristics.

**Years of publication**	**2019–2025**
Countries of studies	**Europe** Germany^13^, ^14^ Italy^15^ The Netherlands^16^ United Kingdom^17^, ^19^ Turkey^18^ **Asia** Taiwan^20^ China^21^, ^22^ Korea^23^ **North America** USA^24^, ^26^, ^27^ Canada^25^

Context of AI used	• Accuracy of AI to detect critical CPR quality parameters and provide real-time feedback^13^, ^14^, ^16^, ^17^, ^20^, ^21^, ^23^ (*n* = 7/47 %) • Use of AI to create personalized training^18^, ^24^, ^25^ (*n* = 3/20 %) • Detection and analysis of dialog segments during and after simulation^15^, ^27^ (*n* = 2/13 %) • Use of AI to create medical teaching illustrations^22^ (*n* = 1/7 %) • AI used in creation of interactive simulations^26^(*n* = 1/7 %) • AI used to answer layperson’s medical questions around cardiac arrest and CPR^19^ (*n* = 1/7 %)

**Table 2:** **Included publications describing Artificial Intelligence used in CPR training.**Appendix 3 gives an overview with more details of the included publications.

**Author, Year, Country**	**Aim, Setting, Design**	**AI Description**	**Main Findings**	**Limitations**
Ecker, 2024, Germany^13^	Assess accuracy of MediaPipe Pose for detecting compression frequency/depth in simulated CPR. Proof-of-principle, 9 archival videos (70/110/140 min^−1^; 35/50/70 mm).	MediaPipe Pose (33 landmarks) integrated into custom app to track CPR provider, estimate frequency/depth.	Frequency correlated well with manikin (*p* < 0.05). Depth only accurate at 110/min with 35–50 mm; higher depths underestimated.	Not approved for clinical use; manikin depth limited; single camera angle; one provider.
Huang, 2024, Taiwan ^20^	Develop and validate SmartCPR, a smartphone app for CPR training/assessment. Experimental validation vs. Laerdal QCPR.	Android phone + TensorFlow Lite + MoveNet; 6 skeletal points tracked; neural network classifies CPR vs. no CPR.	Depth accuracy 84.2 % (≤0.5 cm error); MAE 0.33 cm; no significant difference vs. QCPR. Frequency/posture feedback possible, but not evaluated.	Only depth tested; accuracy affected by hair/backlighting.
Coro, 2022, Italy^15^	Assess workflow to detect ineffective communication in simulation audio. Comparative study of 10 files (79 min), benchmarked vs. 2 experts.	Audio processed for speech energy/intonation, transcribed, keywords extracted via NLP.	Accuracy 64 % (range 37–79 %); *κ* = 0.07–0.46; 11.7 % false negatives; 40 % labeled ineffective vs. 27.5 % gold standard; 60 % time reduction for experts.	Sensitive to noise; no speaker identification.
Sense, 2021, USA^24^	Investigate combining cognitive modeling + ML to predict CPR training outcomes. Secondary analysis of 393 student sessions across spacing/maintenance conditions.	PPE cognitive model; ML models (decision tree, random forest, ridge, lasso) trained on spacing, scores, learner data.	PPE alone MAE 19.5; ML alone worse; ridge/lasso ∼20 MAE; ensemble modestly improved residual predictions.	Complex models harder to interpret; limited added value.
Di Mitri, 2022, Germany^14^	Develop and test CPR Tutor, a multimodal real-time feedback system. Two-phase intervention with expert dataset (*n* = 10) and feedback test (*n* = 10).	Kinect v2 + Myo armband + LSTM; detects 5 indicators (depth, rate, release, arms, body weight); gives audio feedback.	Feedback triggered 16 times; immediate error reduction; metronome most effective; no significant group difference overall.	Small sample; limited evaluation.
Constable, 2025, United Kingdom^17^	Assess deep learning + computer vision for automatic CPR assessment. 53 nursing students, 6 video angles, compared to expert ratings.	Markerless 3D pose estimation + deep learning AQA network to score CPR.	Accurate posture metrics (error < 1); higher error for depth and rate.	Single cohort; depth/rate less reliable.
Di Mitri, 2019, the Netherland^16^	Investigate multimodal data to detect CPR mistakes. Observational study, 14 students (22 sessions).	Multimodal Tutor: ResusciAnne + Kinect + Myo armband; LSTM detects errors in depth, rate, release, arms, body weight.	Accuracy: rate 87 %, depth 72 %, release 74 %; arms 93 %, body weight 98 %.	Not for beginners; 3 participants excluded; rare errors mimicked by 1 subject; setup sensitive.
Ruberto, 2021, Canada^25^	Test adaptive simulation adjusting to cognitive load. Proof-of-concept, 4 participants (10-min AR asthma scenario).	ECG + skin response sensors + deep multitask NN; HoloLens AR adjusted symptoms to cognitive load.	Cognitive load detected in real time; scenario difficulty adapted successfully; valued by participants.	Pilot (*n* = 4); single scenario; predefined distractors.
Liu, 2023, China^21^	Explore action segmentation for CPR instruction. Proof-of-concept, 99 videos with 15 action categories.	PhiTrans model: feature extractor, transformer segmentation, prediction calibrator.	Accuracy >91 % across metrics.	Short-duration actions poorly segmented.
Zhu, 2024, China^22^	Assess DALL·E 3 for generating ECGs and CPR illustrations. Feasibility study.	ChatGPT-4 prompts → DALL·E 3 images (ECG + CPR).	ECGs incomplete (no T waves, artifacts). CPR illustrations clear and usable.	Limited ECG understanding; invalid for teaching ECGs.
Scherr, 2023, USA^26^	Test ChatGPT 3.5 for interactive clinical simulations. Exploratory, 3 scenarios, accuracy reviewed by emergency physician.	ChatGPT 3.5 with iterative prompt refinement for adaptive, interactive simulations.	Created evolving simulations; users made diagnostic/therapeutic decisions.	Accuracy/replicability concerns; no student outcomes.
Ko, 2024, Korea^23^	Develop and validate smartphone-based CPR feedback tool. Observational, mixed participant group.	Galaxy smartphones + manikin; multitask model converts videos into images; feedback on count, depth, release, hand position.	Accurately estimated 4 CPR components; critical-frame method outperformed standard models.	Stationary camera only; compression focus only.
Scquizzato, 2024, Uniterd Kingdom^19^	Evaluate ChatGPT responses to lay CPR/cardiac arrest questions. Mixed-methods, 40 co-produced questions; reviewed by 14 professionals, 16 laypeople.	ChatGPT (Mar 14 preview) generated answers; evaluated for accuracy, clarity, readability, etc.	Median readability 34 (“difficult”); CPR answers rated lower; factually correct but linguistically complex; laypeople rated as good/excellent.	Poor readability; CPR-related answers weaker.
Jones, 2021, USA^27^	Use NLP to analyze transcripts of CPR trial interactions. Secondary analysis of 40 sessions (coached vs. non-coached) in simulated pediatric arrests.	Transcripts processed with NLP, annotated for cues (rate, depth, positive feedback).	Coached teams gave more cues/feedback (*p* < 0.05); fewer questions; depth cues adapted over time.	Non-verbal cues excluded; simulated setting; single mic limited quality.
Molu, 2025, Turkey^18^	Compare AI-based vs. traditional care plan learning in neonatal resuscitation training. Quasi-experimental RCT, 70 nursing students.	ChatGPT-generated neonatal cases used for student care plans; adaptive vs. static control.	AI group scored significantly higher post-test (*p* < 0.05).	Single cohort; ChatGPT (2021 version).

AI applications varied across studies. Several (*n* = 7) focused on the accuracy of AI to detect critical CPR quality parameters and provide real-time feedback, enabling immediate performance improvement.^16^, ^17^, ^19^, ^20^, ^23^, ^24^, ^26^ AI was also applied to create personalized training programs (*n* = 3),^21^, ^27^, ^28^ analyze dialog segments during and after simulations (*n* = 2),^18^, ^30^ develop medical teaching illustrations (*n* = 1),^25^ create interactive simulations (*n* = 1),^29^ and even answer laypersons’ medical questions related to cardiac arrest and CPR (*n* = 1)^22^ (Table 1).

### Accuracy of AI to detect critical CPR quality parameters and provide real-time feedback (*n* = 7^16^, ^17^, ^19^, ^20^, ^23^, ^24^, ^26^)

One study assessed the accuracy of an open-source AI software (MediaPipe Pose Landmark Detection (Version 0.4; Alphabet Inc., Mountain View, California, USA)) in correctly detecting chest compression frequency and depth in nine archival videos of simulated compression-only CPR by one individual. There was one tripod-mounted front-facing camera and only one person in the video frame. The AI yielded similar values for compression frequency on average as the used manikin’s internal software compression log. However, the AI tool tended to underestimate the compression depth.^16^

A study assessed AI-based human posture estimation technology using smartphone images for pose recognition, the TensorFlow Lite (machine learning) for real-time pose estimation, and the MoveNet (AI-based pose detection model), which recognizes 6 key skeletal points of the person performing CPR in real time. A trained neural network discerned between CPR and no CPR phases (SmartCPR system). Chest compression depth was compared with Laerdal’s QCPR system (Laerdal Medical, Stavanger, Norway). The SmartCPR system showed a lower mean absolute error than QCPR, however with no significant difference between QCPR and SmartCPR. The system could be obscured by long hair or backlighting if joint positions could no longer be accurately detected.^23^

Another study used the CPR Tutor, which is a real-time feedback system using recurrent neuronal networks (specifically Long Short-Term Memory networks) to analyze and classify multimodal data collected from a Kinect v2 sensor (Microsoft, Redmond, Washington, USA) and a Myo electromyographic armband (North Inc., Kitchener, Ontario, Canada). Compression release, depth, rate, arms locked, and body weight were assessed. The system was trained with 10 experts’ CPR performances including mistakes, and then tested with 10 new participants, comparing a feedback-enabled mode versus a no-feedback mode. The system provides real-time audio feedback to help correct performance. The error rates of assessed CPR performance decreased after feedback prompts. A metronome as audio feedback was the most effective feedback intervention. However, no significant difference in mistake frequency between the control and feedback groups was found.^17^

Another study used baseline data and CPR performance metrics from the Laerdal QCPR ResusciAnne manikin (Laerdal Medical, Stavanger, Norway) with a multi-sensor system (Microsoft Kinect for tracking body position (Microsoft, Redmond, Washington, USA), and a Myo armband (North Inc., Kitchener, Ontario, Canada) for collecting electromyogram and accelerometer information). They trained Long Short Term Memory Networks to detect common CPR mistakes with 11 participants, resulting in compression depth (72 % accuracy), compression rate (87 % accuracy), and compression release (74 % accuracy). These three CPR quality indicators closely matched the ResusciAnne manikins’ values. Additional mistakes investigated were improper arm locking (93 % accuracy) and body weight usage (98 % accuracy). However, for these additional mistakes, a separate dataset was created with one participant mimicking the 2 errors.^19^

One study assessing how deep learning and computer vision techniques can automatically assess CPR performance with 53 participants from nursing and midwifery showed mixed results. Markerless pose estimation (a technique in computer vision using machine learning and deep learning) was used to capture 3D positions of participants’ joints from different angles with 6 video cameras, but no sensors or markers were used. A deep learning network designed for automatic action quality assessment (AQA) was trained to assess CPR performance against predefined criteria. Compared with manual assessments, the AQA consistently exhibited significantly lower errors in evaluating hand, arm, and shoulder positions, but higher error rates in compression depth and rates.^20^

One study investigated whether CPR instruction could be enhanced with action segmentation. Ninety-nine 2-min videos of participants performing CPR in a standard green screen laboratory were used. The model was called PhiTrans using a Video Features Extractor, Action Segmentation Executor, and Prediction Refinement Calibrator. The PhiTrans model was performing well on all metrics, surpassing 91 %.^24^

Another study validated a deep-learning-based CPR training tool providing real-time feedback using smartphone-recorded chest compression videos. The model converts the chest compression videos into images, provides feedback on chest compression count, depth, chest release and incorrect hand position via multitask based optimization. The model successfully and effectively estimated the CPR components. Converting videos into images improved detection. Various backgrounds were used in different indoor locations, including people present in the background. The model used stationary camera footage.^26^

### Use of AI to create personalized training (*n* = 3^21^, ^27^, ^28^)

One study investigated whether combining cognitive modeling and machine learning can improve the predictive accuracy of personalized training schedules for CPR skill acquisition and retention. The authors used predictive performance equation (PPE), and four different machine learning models, and data from a previous study evaluating a cognitive model was included. The machine learning models tested would not have resulted in better predictions then the PPE alone. There were modest improvements in predictive accuracy for models, in which machine learning predicted the prediction errors (e.g., residuals) of the standalone cognitive model.^27^

Another study tested a novel simulation platform capable of adjusting the difficulty based on real-time cognitive load measurement of participants. This was a pilot study with 4 participants using electrocardiography and galvanic skin response sensors, and a deep multitask neural network classifying cognitive load as high or low. Cognitive load was artificially increased during the simulation with using distractors. An augmented reality patient (displayed via Microsoft HoloLens, Microsoft, Redmond, Washington, USA) was used. Measurement of cognitive load was successful, simulation difficulty was adapted successfully. There were no other outcomes reported.^28^

A RCT with a pre-post-test control group compared the efficacy of an AI-based care plan learning strategy with standard training techniques to determine how it affects nursing students’ learning results in newborn resuscitation. Both groups received 4-week neonatal resuscitation training prior. The experimental group received AI care plans, the control group received traditional instructions. The AI‐based care plans allowed students to engage with a variety of neonatal resuscitation challenges, providing a more adaptive and customized learning experience. The traditional care plans were static and did not change based on individual student performance. Both groups pre‐test scores were similar. In the post‐test, the AI‐based group outperformed the traditional group (*p* < 0.05).^21^

### Detection and analysis of dialog segments during and after simulation (*n* = 2^18^, ^30^)

One study assessed an automatic workflow for the detection of dialog segments with potentially ineffective communication between team members. Ten historical recorded audio files were used under different noise conditions and team and gender compositions. The workflow labels segments with potentially ineffective communication due to alterations of speech energy and intonation. Segments are then transcribed, words are extracted. Two content experts validated the accuracy of the system. Overall accuracy was 64 %, with a fair agreement between the model and the experts. False negatives (unlabelled potentially ineffective communication) were 12 %; AI labeled 40 % as potentially ineffective, experts only 28 %. However, the model was able to reduce the time required to analyze the audio recordings by 60 %. The workflow was unable to distinguish team members.^18^

Another study used NLP to analyze transcripts of team interactions from a published RCT of audio data comparing a coached vs. a non-coached trial arm. After transcribing audio data, NLP split it into sentences, annotated as questions or statements. Sentences were split into words, compared against task-specific verbal cues; if a word matched a CPR term, it was annotated with the corresponding label (compression depth, rate, positive feedback). All annotations were reviewed manually. NLP was successfully used to analyze transcripts of team interactions. Coached groups had more unspecified positive, rate directional, depth-positive, and depth-directional utterances per minute. The frequency of depth cues from coaches adapted over time, indicating they adjusted their feedback as CPR progressed.^30^

### Use of AI to create medical teaching illustrations (*n* = 1^25^)

This study used ChatGPT-4 (OpenAI, San Francisco, USA) to generate a detailed text prompt specifying the parameters of a standard 12-lead ECG, which was then input into DALL-E 3. DALL-E 3 is a text-to-image generation model also developed by OpenAI. It was used to generate images of a 12-lead electrocardiogram and resuscitation-related visuals based on the text prompts provided by ChatGPT-4. The ECG DALL-E 3 produced could not be considered a standard electrocardiogram as it contained several interfering waves; the CPR teaching illustrations were satisfactory.^25^

### AI used in creation of interactive simulations (*n* = 1^29^)

This study evaluated ChatGPT 3.5’s ability to perform interactive clinical simulations. It was judged as being capable of creating interactive clinical simulations by a board-certified emergency physician.^29^

### AI used to answer layperson’s medical questions around cardiac arrest and CPR (*n* = 1^22^)

This study evaluated the ChatGPT AI model’s (free research preview, March 14 version) ability to provide answers to layperson’s questions about cardiac arrest and CPR. Forty questions were used. Responses were assessed by laypersons and healthcare providers. Answers were judged as largely factually correct by healthcare providers, and good or excellent by laypersons, despite being linguistically difficult according to the Flash Reading Ease score.^22^

This scoping review mapped the use of AI within CPR training and identified 15 relevant publications between 2019 and 2025. AI has been applied to several areas of CPR training, including automated detection of CPR quality parameters, provision of real-time feedback, creation of personalized training schedules, analysis of communication patterns during simulation, generation of educational illustrations, and answering medical questions of laypeople. Interestingly, all studies except of one were not actually involving AI directly for actual training but rather as proof-of-concept in an artificial environment. This large number of proof-of-concept studies calls for more comparative research between AI and traditional CPR training. Overall, the field of AI in CPR training is still emergent. Most studies did not involve AI being used directly for hands-on training, but only explored potential applications and feasibility.

We noticed while reading the manuscripts that most included studies had low quality of evidence. Most of the included studies were observational, single-center studies with lacking control groups and underpowered which itself carries a significant risk of bias. Furthermore most studies were proof-of concepts,^16^, ^17^, ^18^, ^19^, ^20^, ^22^, ^23^, ^24^, ^25^, ^26^, ^27^, ^28^, ^29^, ^30^ with only one randomized trial comparing an AI intervention with a traditional educational method.^21^

Seven studies evaluated the accuracy of AI to detect critical CPR quality parameters and provide real-time feedback.^16^, ^17^, ^19^, ^20^, ^23^, ^24^, ^26^ All were proof-of concept studies. Most studies used static videos to train their AI. The studies showed mixed results in being comparable to standard CPR quality parameter detection via training manikin systems. Further research is necessary to improve these AI tools to be able to capture real simulation training sessions from a moving camera and not in a static environment. The studies reported only specific CPR components, such as chest compression metrics (depth and rate), whereas basic and advanced life support practice includes scenario practice with multiple sequence of actions, handling of contextual factors, and teamwork which are of importance in real-life resuscitation. However, AI seems promising for providing real-time feedback to course participants, but further development and comparative evaluation are necessary.

Three studies evaluated AI in creating personalized training opportunities.^21^, ^27^, ^28^ Two studies were proof-of-concept studies which successfully created personalized training opportunities and training adaptations. The third study, with a quasi-experimental design, compared AI-based care plan scenarios with traditional instructor-prepared case stories in neonatal resuscitation training for nursing students and demonstrated superior learning outcomes in the AI-supported group. This is the first study showing, in a standardized educational setting, the potential benefit of integrating AI generated learning materials into CPR training.

Two studies evaluated AI in the detection and analysis of dialog segments in team-based simulations.^18^, ^30^ Although still with moderate accuracy, this technology offers intriguing possibilities for automated debriefing and identifying communication breakdowns. Further studies are needed to implement the use of AI in the analysis of dialog segments, ideally in real-time, before feedback is delivered to the participants.

AI for generating teaching illustrations, focusing on ECGs and CPR instruction illustrations, showed mixed results. The used AI model seems to be less accurate in creating a standard ECG; however, it was successful in creating teaching illustrations.^25^ AI was used successfully to create interactive simulations. It was judged capable by a board-certified emergency physician.^29^ AI’s capacity to answer layperson questions around cardiac arrest and CPR was judged as largely correct by healthcare providers and as good or excellent by laypersons.^22^ It seems that current large language models are already capable of answering laypersons’ CPR questions.

### Limitations

Due to the heterogeneity of the found studies and the diverse application of AI, we were unable to perform a systematic review, as only one study compared AI with traditional CPR teaching. Therefore, we cannot draw definitive conclusions about the efficacy of specific AI interventions. However, the rapidly evolving AI technologies warrant close observation and evaluation of potential cutting-edge developments that emerge. Given the rapid evolution of AI technologies, new developments in this field are likely to emerge soon; therefore, an update of this review within a reasonable time frame will be important to capture future developments.

### Knowledge gaps and future research

The existing literature suggests that emerging AI applications could complement traditional instructor-led CPR training by providing individualized, real-time feedback, enhancing accessibility, and supporting learning. Despite the promising applications, significant knowledge gaps exist, highlighting several crucial areas for future development and research. There is definitely a lack of high-quality RCTs demonstrating the effectiveness of AI-based CPR training over traditional training methods. Most studies identified were proof-of-concept studies, establishing feasibility rather than efficacy. Future research must move beyond feasibility to rigorously compare AI-enhanced training with traditional methods, focusing on skill acquisition, retention, and ultimately, real-world patient outcomes. In this review, we categorized AI broadly; in the future, a deeper understanding of which specific AI methods are most effective for which aspects of CPR training will be needed.

Uncertainty exists due to the lack of research on the ethical implications of AI in medical education. Variability in underlying algorithms may privilege or disadvantage certain approaches to CPR training, learner groups, or instructors. Future work should extend beyond technical refinement to include evaluations of cost-effectiveness and societal impact of AI-based CPR training.

## Conclusion

AI has potential to transform CPR training. Evidence emerges for enhanced real-time CPR skill performance feedback, personalized learning, and improved debriefing through the analysis of dialog segments from CPR training and simulation. Large language models provide correct information about cardiac arrests and CPR. Nevertheless, this remains an emergent field, the evidence is characterized by proof-of-concept studies and a lack of high-quality comparative evidence of efficacy of AI-based CPR training compared to traditional teaching methods, assessing educational and patient outcomes.

## Other disclosures

None.

## Ethics committee review

Not applicable.

## Disclaimers

None.

## CRediT authorship contribution statement

**Timo de Raad:** Writing – review & editing, Writing – original draft, Project administration, Methodology, Investigation, Formal analysis, Conceptualization. **Olfa Chakroun-Walha:** Writing – review & editing, Investigation, Formal analysis. **Brenna Leslie:** Data curation. **Robert Greif:** Writing – review & editing, Methodology, Formal analysis, Conceptualization. **Sabine Nabecker:** Writing – review & editing, Writing – original draft, Project administration, Methodology, Investigation, Formal analysis, Conceptualization.

## Funding

None.

## Declaration of competing interest

The authors declare the following financial interests/personal relationships which may be considered as potential competing interests. SN, and RG are members of the ILCOR EIT Task Force (RG is chair). RG is ERC Director of Guidelines and ILCOR, RG is Editorial Board member of Resuscitation Plus. TDR and SN are members of the ERC SEC-IES (TDR is co-chair). SN is editorial board member of BMC Medical education. TDR, OCW, RG and SN are authors of the European Resuscitation Council Guidelines 2025: Education for Resuscitation.
