# Supplementary material for: Artificial Intelligence in cardiopulmonary resuscitation training – A scoping review
Source: Resusc Plus. 2025 Nov 22;27:101175. doi: 10.1016/j.resplu.2025.101175 (PMC12722975; doi:10.1016/j.resplu.2025.101175)
Supplement: Supplementary Appendix 2 [file mmc2.pdf]

Appendix 2 - Artificial Intelligence in cardiopulmonary resuscitation training – a scoping review. Search strategy.

Ovid MEDLINE(R) ALL <1946 to July 02, 2025>

1 exp Resuscitation/ or Advanced cardiac life support/ or exp Heart arrest/ or Defibrillators/ 155017

2 ((chest* or rescue* or resuscitat*) adj2 (cardio* or pulmonar* or compress* or breath* or cardiac* or mouth*)).ti,ab. 32051

3 (resuscitat* or defibrillat*).ti,ab. 110185

4 ((cardiac* or cardio* or heart*) adj2 massag*).ti,ab. 1471

5 (cpr or aed or bls or acls or bcls or ohca).ti. 4036

6 ("code blue" or (code adj1 blue)).ti,ab. 380

7 (life support* adj2 (advanc* or basic* or cardiac*)).ti,ab.7401

8 ((death* or arrest*) adj2 (cardiac* or cardio* or heart*)).ti,ab. 115044

9 asystol*.ti,ab. 4977

10 (artificial* adj2 (respirat* or ventilat*)).ti,ab. 7820

11 or/1-10 296635

12 Artificial intelligence/ or Neural Networks, Computer/ or Computer Heuristics/ or Expert systems/ or Algorithms/ or Natural Language Processing/ or exp Machine learning/ or Latent class analysis/ or Sentiment analysis/ or Random forest/ or Computing methodologies/ or Support Vector Machine/ or exp Virtual reality/ or Augmented reality/ 486329

13 (AI or VR).ti. 15699

14 (intelligence adj1 (artificial* or computation* or machine* or computer*)).ti,ab. 66073

15 (learning* adj1 (machine or transfer* or deep or hierarchical or active or inductive or semi-supervised or teacher or semisupervised or "labeled data")).ti,ab. 207739

16 ("machine learn*" adj1 (supervised or unsupervised)).ti,ab. 5268

17 ((machine or network*) adj1 "support vector").ti,ab. 26567

18 ("ai" adj2 driven adj2 adapt* adj2 learning).ti,ab. 4

19 (natural* adj1 language* adj1 process*).ti,ab. 10056

20 ((virtual* or realit*) adj1 (augment* or mixed or virtual*)).ti,ab. 200021

21 (comput* adj1 (method* or "high performance" or heuristic* or reasoning or "vision system*" or "knowledge acquisition")).ti,ab. 32646

22 ("expert system*" or "fuzzy logic*").ti,ab. 6258

23 ((comput* or connectionist*) adj1 ("neural network*" or model*)).ti,ab. 48839

24 (algorithm* or avatar* or haptic*).ti,ab. 445717

25 ((mining* or analys* or classification) adj1 (opinion or sentiment*)).ti,ab. 2397

26 ((analys?s* or model*) adj1 ("latent variable" or "structural equation" or "latent class*" or "probabilistic latent")).ti,ab. 48467

27 (random* adj1 forest*).ti,ab. 35827

28 or/12-27 1146821

29 Teaching/ or Learning/ or Education/ or Curriculum/ or exp Simulation training/ 242848

30 (educat* or learn* or teach* or taught* or program* or curricul* or train* or workshop* or techni* or method* or pedagog* or instruct* or phenomenograph* or "short term course" or simulat*).ti. 1991582

31 ((learn* or train*) adj1 (interact* or simulat*)).ti,ab. 9094

32 or/29-31 2078273

33 11 and 28 and 32 1565

34 limit 33 to (guideline or meta analysis or practice guideline or "review" or "scoping review" or "systematic review") 123

35 33 not 34 1442

36 limit 33 to yr="2024 -Current" 370

37 limit 36 to (guideline or meta analysis or practice guideline or "review" or "scoping review" or "systematic review") 22

38 36 not 37 348
