# Supplementary material for: Artificial Intelligence in cardiopulmonary resuscitation training – A scoping review
Source: Resusc Plus. 2025 Nov 22;27:101175. doi: 10.1016/j.resplu.2025.101175 (PMC12722975; doi:10.1016/j.resplu.2025.101175)
Supplement: Supplementary Appendix 3 [file mmc3.pdf]

Appendix 3 – Table with an extensive description of the included publications describing Artificial Intelligence used in CPR training

Author, Year, Country	Aim, Setting, Study design	Description of Artificial Intelligence Used	Main Findings	Limitations, Comments
Ecker, 2024,<br><br>Germany[13]	Assess accuracy of the open-source AI software (MediaPipe Pose Landmark Detection, in correctly detecting compression frequency and depth in video footage of simulated CPR<br><br>Nine archival 60s videos of simulated compression-only CPR performed by one individual on a manikin with varying compression frequencies (70, 110, and 140/min) and depths (35, 50, and 70 mm).<br><br>Experimental proof-of-principle study<br><br>Comparison with manikin's internal software compression log.	MediaPipe Pose Landmark Detection (Version 0.4; Alphabet Inc., Mountain View, California, USA) is an open-source machine-learning tool developed by Google. The software is customizable and allows landmark detection of a body pose using a machine learning model to detect the presence of human bodies within an image frame, and a second machine learning model to locate landmarks on the bodies.<br><br>It tracks 33 body landmark locations, creating a skeletal representation of the human body.<br><br>It was incorporated into a custom web-based software by NEOANALOG (Schmitz & Rabe GbR, Cologne, Germany) to identify a person providing CPR and giving real-time feedback by analyzing the body tracking data. Changes in hand- and shoulder position were used to calculate chest compression frequency and depth.	There was a significant correlation between compression frequency recorded by the AI tool and the manikin registered data for all compression frequencies, regardless of the compression depth ($p<0.05$).<br><br>Bland-Altman plots showed that there was a relatively small difference in compression frequency measured by AI and manikin, which indicated both methods yield similar values on average. There was moderate variability of the Standard Deviation of Differences, but most data points fell within +/- 1.96 standard deviations.<br><br>Comparison of compression depth showed only significant correlation for compression frequencies of 110/min with compression depths of 35 and 50 mm, all other compression depths were not accurately determined by the AI tool, which tended to underestimate the compression depth.<br><br>Bland-Altman plots for compression depths (35 and 50 mm) show that the mean difference of the AI-based measurement tend to provide lower values than the manikin. Higher compression depths were more underestimated than lower compression depths. Standard deviation of differences was moderate, and only a few data points fell outside the +/- 1.96 standard deviations.	Software is not approved for medical purposes, this was a proof-of-principle study.<br><br>Statistical testing on compression depth of 70 mm was not performed, as the manikins maximum compression depth was limited to 65 mm, because of technical limitations of the manikins compression coil.<br><br>The AI was tested from one fixed (tripod-mounted) camera angle (front-facing).<br><br>There was only 1 person in the video frame.
Huang, 2024, Taiwan[20]	Develop and test a smartphone-based application for training and assessment of CPR (SmartCPR)<br><br>System development and experimental validation.<br><br>Chest compression depth comparison with Laerdal QCPR system.	AI-based human posture estimation technology integrated into a smartphone application to create the SmartCPR system, which automatically detects and assesses chest compressions during CPR.<br><br>An android phone is used to capture images for pose recognition.<br><br>TensorFlow Lite (version of the TensorFlow machine learning framework optimized for mobile devices) is used for real time pose estimation.<br><br>MoveNet (AI based pose detection model) recognizes 6 key skeletal	Chest compression depth:<br>- In comparison to QCPR, SmartCPR showed a lower mean absolute error (0.32517) than QCPR. Mean absolute error represents the average discrepancy between compression depth measured by two methods.<br>- Accuracy was 84.2%, data was counted as accurate if compression depth error was <= 0.5 cm.<br>- Insufficient evidence of a significant difference between QCPR and SmartCPR ($p=0.05769$).	The system is able to provide real-time feedback on depth, frequency, and posture errors, which traditional devices do not address, but the manuscript does not provide any data on compression frequency or posture errors.<br><br>The system's accuracy is potentially impacted by long hair or backlighting, which can obscure joint positions and lead to misjudgments in posture

		points of the person performing CPR in real time.<br><br>Trained neural network classifier weights are used to discern between CPR and no CPR phases in the CPR cycle.		detection.
Coro, 2022, Italy[15]	Assess an automatic workflow for detection of dialogue segments of a simulation session with potentially ineffective communication between team members.<br><br>Ten historical recorded audio files, total duration 79 min 5 s under different noise conditions and team and gender composition<br><br>Comparison with two independent context experts.<br><br>Comparative study.	Workflow processes one-dimensional audio-signal recorded by a microphone. It labels segments containing potentially ineffective communication due to alterations of speech energy and intonation.<br><br>The detected segments are transcribes using an automatic speech recognizer (ASR).<br><br>The transcribed text is further processed to extract words containing enough semantic information to understand the dialogue contents.	Overall accuracy ranged from 37-79%, overall 64%.<br><br>Agreement between to unsupervised model and the gold standard ranged from 0.066 to 0.455. Agreement was fair over all recordings.<br><br>The percentage of unlabelled potentially ineffective communication (the false negatives) was 11.7%.<br><br>The actual percentage of audio segments containing potentially ineffective communication was 27.5%, whereas 40% was automatically labelled as potentially ineffective.<br><br>The viability of the detected keywords, measured as the percentage of gold standard words contained in the word cloud was 59%.	The workflow required ca. 10 s to process a 10 min recording.<br><br>The workflow reduced the time required for experts to analyze audio recordings by 60%.<br><br>The workflow was poorly sensitive to higher noise levels – when compared to evaluating transcripts rather than audio. It was rather more sensitive to frequency of the interactions, intonation and audio volume.<br><br>The workflow does not identify individual speakers making it difficult to distinguish team members.
Sense, 2021, USA[24]	Investigate whether combining cognitive modeling and machine learning approaches can improve the predictive accuracy of personalized training schedules for CPR skill acquisition and retention.<br><br>Data from previous study evaluating a cognitive model:<br><br>Historical data from 393 CPR training sessions, nursing students trained in 4 different acquisition conditions, 4 CPR training sessions spaced by 1 day, 1 week, 1 month, or 3 months; 3 different maintenance training schemes for 1 year at intervals of 3, or 6 months, or personalized prescribed by the cognitive model<br><br>Resuscitation Quality Improvement (RQI) system	Predictive performance equation (PPE): set of nested mathematical equations that capture findings in the cognitive science literature associated with the temporal dynamics of human learning and forgetting. In the initial study PPE was fit separately to each participant's history of compression and ventilation scores.<br><br>Four different machine learning models were used, they were trained to predict the score or the PPE's residuals. Single decision tree, random forest (ensemble of decision trees), ridge regression and lasso.<br><br>The models used input features from the training data, such as time between training sessions, previous scores, and learner characteristics, to make their predictions.	The original PPE alone yielded a mean absolute error of 19.5.<br><br>All machine learning approaches yielded average mean absolute errors larger than 19.5 when applied alone, which suggests that the machine learning models tested here—if used by themselves—would not have resulted in better predictions overall.<br><br>The ridge and the lasso regression performed well on average (MAE 20.2 and 20, respectively).<br><br>The average MAE for the original PPE is hardly affected by adding any of the machine learning models to predict its residuals.<br><br>The decision trees seem to worsen performance more often than not.<br><br>Although the original PPE yields the lowest overall average MAE,	More complex models might be harder to interpret.

			both the profile and skill variants achieve better average ranks. This suggests that if machine learning models are leveraged to predict PPE's residuals, more constrained variants of PPE tend to perform better.<br><br>Therefore, there are modest improvements in predictive accuracy for ensemble models, in which machine learning models predict the prediction errors (i.e., residuals) of the standalone cognitive model.	
Di Mitri, 2022, Germany[14]	Development and evaluation of a CPR tutor, a real-time multimodal feedback system for CPR training.<br><br>The primary objective is to explore how multimodal data (collected from Kinect sensors and Myo armbands) can improve learning outcomes in CPR training providing feedback to correct errors during training. The study also investigates how machine learning models can help detect mistakes and deliver personalized, adaptive feedback during simulation-based medical training<br><br>Two-phase intervention study:<br><br>Phase 1 - Expert Data Collection - A group of 10 experts was asked to perform four sessions of 1-minute duration each, 2 sessions each included mistakes, such as not locking arms or not using body weight.<br><br>This phase aimed to create a balanced dataset of correct and incorrect CCs for training the CPR Tutor's machine learning model.<br><br>Phase 2 - Feedback Intervention: A new group of 10 participants, each performing two sessions of 1-minute duration:<br><br>One session with feedback enabled, One session without. In alternating order.	The CPR Tutor, is a real-time feedback system using recurrent neural networks, specifically Long Short-Term Memory networks, to analyze and classify multimodal data collected from a Kinect v2 sensor and a Myo electromyographic armband. The system was designed to automatically recognize and assess the quality of chest compressions during CPR training across five performance indicators: compression release, depth, rate, arms locked, and body weight. The system provides audio feedback immediately after detecting a mistake to help correct performance.	In the feedback intervention phase the CPR Tutor feedback function was enabled in 10 sessions, It was fired 16 times with 2 release, 5x depth, 5x rate, 1x arms locked and 3x body weight. The error rates of all five target classes decreased soon after feedback is prompted. The audio feedback using the metronome sound correct was the most effective feedback intervention. There was no significant difference in mistake frequency between the control and feedback group.	Field study with 10 participants.
Constable, 2025, United Kingdom[17]	Assess how deep learning and computer vision techniques can be used to automatically assess CPR performance and avoid the subjectivity of human assessments.<br><br>53 participants from Department of Nursing and Midwifery	The AI intervention integrates markerless pose estimation to capture 3D positions of a participant's joints from different angles in the video (6 camera angles, no sensors or markers needed) and a deep learning network designed specifically for automatic action quality assessment (AQA). The network is trained to assess the quality of CPR performance against predefined criteria. It produces ratings for various aspects of CPR, thus providing an objective, automated	When comparing this method with manual assessments,<br><br>automatic AQA consistently exhibited significantly lower error in evaluating hand, arm, and shoulder positions (error margings below 1) – posture related metrics. However, the AQA exhibited higher error rates in the compression depth and compression rate items – interaction between posture and the manikin.	The CPR skills performance database (collected for the purpose of this research,<br>hosted at UK Data Service's ReShare Repository) contains videos from 40 participants recorded from 6 different angles, allowing for 3D reconstruction

		evaluation of the participant's skill level.		for movement analysis. The video footage is accompanied by quality ratings from 2 experts, participants' self-reported confidence and frequency of performing CPR, and the demographics of the par-<br>ticipants.
Di Mitri, 2019, the Netherlands[16]	Investigate to what extent multimodal data can be used to detect mistakes during CPR training.<br><br>Observational study<br><br>Laerdal SimPad SkillsReporter to log CPR performance metrics.<br><br>Advanced medical and medical dentist students.<br><br>Each participant (14) completed two 2-minute sessions of chest compressions, separated by a 5-minute break, for a total of 22 recorded sessions (3 participants were excluded see limitations).	A Laerdal QCPR ResusciAnne manikin (baseline data and CPR performance metrics) was complemented with the Multimodal Tutor for CPR, a multi-sensor system consisting of a Microsoft Kinect for tracking body position and a Myo armband for collecting electromyogram and accelerometer information.<br><br>The Machine Learning Model used was a type of recurrent neural network, Long Short Term Memory Networks (LSTM), which is able to learn over long sequences of data. Each iteration leaves a footprint, which is used to calculate the following iterations.<br><br>Trained to detect common CPR mistakes: Compression rate, depth, and release. Additional mistakes: Improper arm locking and body weight usage.	The Multimodal Tutor for CPR accurately detected common CPR training mistakes:<br><br>Compression rate: 87% accuracy.<br><br>Compression depth: 72% accuracy.<br><br>Compression release: 74% accuracy.<br><br>The system's performance closely matched the ResusciAnne manikin's baseline, validating its reliability.<br><br>Additional CPR mistakes typically only detectable by human instructors (CAVE: see limitations):<br><br>Arm locking: 93% accuracy.<br><br>Body weight usage: 98% accuracy.	Setup not suitable for beginners, rather participants with prior training knowledge.<br><br>Data from 3/14 participants was discarded due to insufficient quality – either caused by faulty Myo readings or incorrect booting of the Learning Hub.<br><br>For the additional mistakes, an additional data set consisting of five sessions with one participant mimicking the 2 mistakes was used, because none of the initial participants made either of those mistakes.<br><br>The system's accuracy and performance are dependent on a consistent experimental setup.
Ruberto, 2021, Canada[25]	To test a novel simulation platform: to measure cognitive load using artificial intelligence algorithms in real time and modulate the difficulty of the simulation based upon the results.<br><br>Proof of concept study with an experimental design.<br><br>2 board-certified emergency physicians, 2 medical students<br><br>10-minute pilot trial<br><br>Post simulation survey	Adaptive simulation platform that adjusted difficulty based on real-time cognitive load measurement. Using electrocardiography and galvanic skin response sensors, a deep multitask neural network classified cognitive load as high or low. The simulation then dynamically modified the severity of an augmented reality patient's symptoms (displayed via Microsoft HoloLens) to match the participant's cognitive capacity.	Successful measurement of cognitive load in real time through physiological signals. The simulation difficulty was successfully adapted to the participant's cognitive load, which was reflected in changes in the AR patient's symptoms. Participants found the novel adaptive simulation platform to be valuable in supporting their learning.	Pilot study (n=4)<br><br>Scenario used: Asthma exacerbation<br><br>5 distractors to increase cognitive load: ECG displaying sinus tachycardia, a blood glucose level of 14 mmol/L, a home medication list, an emergency medical service patch call, and a simultaneous code blue in the emergency department.

Liu, 2023, China[21]	To investigate how CPR instruction could be elevated with action segmentation<br><br>Proof of concept study<br><br>99 2-min videos of participants performing the whole process of CPR in a standard green screen laboratory environment containing up to 15 action categories (e.g., preparing to start, confirming environment, ensuring safety, etc.)	Integrated model, called PhiTrans, especially applied for CPR action segmentation, including three integral modules: Video Features Extractor (automatically segments CPR training videos at the frame level, prompt-based feature extractor), Action Segmentation Executor (transformer for contextual action segmentation), and Prediction Refinement Calibrator (regression module for refining predictions with the goal to pinpoint key CPR steps and potential issues.	The PhiTrans model is adequate to approach the challenge of assisting CPR instruction, performing well on all metrics used surpassing 91.0%.	PhiTrans struggles with short-duration actions, leading to redundant segments in the segmentation results.
Zhu, 2024, China[22]	Is the DALL-E 3 model able to generate medical teaching illustrations, specifically focusing on creating 12-lead ECGs.<br><br>Feasibility study	Used AI: First ChatGPT-4 (A language model developed by OpenAI) designed to generate human-like text based on given prompts was used to generate a detailed text prompt specifying the parameters of a standard 12-lead ECG, which was then input into DALL-E 3. Also, a didactic illustration of CPR techniques for BLS was requested.<br><br>DALL-E 3 is text-to-image generation model also developed by OpenAI. It uses natural language descriptions to create images that visually represent the given text prompts. In this study, DALL-E 3 was used to generate images of a 12-lead ECG and resuscitation-related visuals based on the text prompts provided by ChatGPT-4.	DALL-E 3 was able to generate an image containing visual features corresponding to some basic elements of ECG tracing, including P waves and QRS complexes. However, the ECG it drew could not be<br><br>considered a standard 12-lead ECG, as it failed to produce obvious T waves and contained several interfering waves.<br><br>The two CPR teaching illustrations drawn by DALL-E 3 were satisfactory, with the requirements in the prompt almost fully presented in the images. The emphasized steps were also shown separately in small<br><br>images, and the core chest compression technique of CPR was clearly illustrated.	The AI evaluated in 2024 does not yet<br><br>have a robust enough understanding of ECG physiology and waveform characteristics to produce valid<br><br>examples.
Scherr, 2023, USA[26]	To test ChatGPT 3.5's ability to perform interactive clinical simulations. The study explores ChatGPT's ability to assist students in forming diagnostic and therapeutic decisions while receiving real-time feedback.<br><br>Exploratory study to create and run 3 simulations from beginning to completion<br><br>Medical accuracy was assessed by a board-certified emergency physician	The AI tool used in the study is ChatGPT 3.5, a generative language model developed by OpenAI. A stepwise approach was employed to refine simulation prompts iteratively, ensuring that ChatGPT could effectively create and run the desired clinical simulations. Researchers tested and modified the initial prompts based on the model's performance, adjusting the instructions to specify the type of scenario, the need for free-response questions, and ensuring the scenario adapted to user input. Several test simulations were run, and the prompts were refined each time based on the output.	ChatGPT 3.5 is capable of creating interactive clinical simulations. The user can request additional investigations. The simulations can evolve to cover multiple problems. Users can make all the diagnostic and therapeutic decisions, rather than having many key details already fleshed out in question stems (e.g., Board Exam questions). The user must recall and apply correct information rather than just recognize it.	Clinical scenarios: ACLS, ICU scenarios (pneumonia, sepsis)<br><br>The simulations evolve and unfold based on the user's actions.<br><br>There were concerns about simulation accuracy and replicability.<br><br>Study does not evaluate student satisfaction or educational outcomes.
Ko, 2024, Korea[23]	Develop and validate a low-cost, deep learning-based CPR training tool that provides real-time feedback using smartphone-recorded chest compression videos.<br><br>Observational study<br><br>Study subjects included the general public, emergency	Videos were taken using the Laerdal Resusci Anne QCPR manikin, and the front cameras of Galaxy S10, Galaxy S10+, and Galaxy Note9 smartphones.<br><br>The used model converts chest compression (CC) videos into appropriate images, and provides feedback on four core	The study successfully and effectively estimated four representative high-quality CPR components using multitask learning.<br><br>The critical-frame-based method outperformed existing video-based models in CPR video analysis. Converting videos into	To ensure generalizable performance, data collection was carried out against various backgrounds in different indoor locations. This process encompassed a variety of

	responders, students, and medical practitioners.	components of high-quality CPR: CC count, maximum compression depth of CCs, complete chest release following compression, and incorrect hand positions of CC via multitask based optimization. To reduce the complexity of video processing, the authors conducted a composite-image-based evaluation for time-independent tasks.	images improved the detection of hand positioning and compression quality, addressing challenges faced by conventional video-processing models.	environmental conditions, including<br><br>those with people present in the background.<br><br>The model was developed using stationary camera footage, which may limit its accuracy when analyzing videos captured with a moving camera.<br><br>The model is only focussed on chest compressions.
Scquizzato, 2024, Uniterd Kingdom[19]	The study aimed to evaluate the accuracy, clarity, relevance, comprehensiveness, and readability of ChatGPT's responses to laypeople's questions about cardiac arrest and CPR.<br><br>Mixed-methods study<br><br>A list of 40 questions was co-produced with members of Sudden Cardiac Arrest UK covering all aspects of cardiac arrest and CPR.<br><br>Responses were assessed by 14 healthcare professionals for accuracy, and by healthcare professionals and 16 laypeople for clarity, readability relevance, comprehensiveness, and overall value using a 5-point scale from 1 poor to 5 excellent. Readability was analyzed with a scoring tool. Statistical tests compared professional and lay evaluations.	ChatGPT AI model (free research preview, March 14 version), a large language model developed by OpenAI was asked to provide answers using each question as input.	Median readability score calculated with the Flesh Reading<br><br>Ease score was 34 or 'difficult' (IQR 26–42).<br><br>Answers to CPR-related questions consistently received a lower score by both professionals and laypeople, across all parameters.<br><br>Generated answers were evaluated by professionals as largely factually correct and rated as good or excellent by laypeople, despite being linguistically difficult according to the Flesh Reading Easy score.	
Jones, 2021, USA[27]	The study used natural language processing (NLP) to analyze transcripts of team interactions during a published randomized trial, aiming to provide insights into how verbal cues and feedback contribute to the improvement of CPR quality in a simulated setting.<br><br>Secondary analysis of audio data from a prospective randomized clinical trial, which aimed to investigate the impact of a CPR coach on the quality of CPR performed during simulated pediatric cardiac arrests.<br><br>2 min preparatory period, followed by 18 min CPR scenario. 40 sessions, 20 in each trial arm (coached vs.	The audio of the video tapes was first transcribed via GoTranscript.com (Premium features). Transcripts were manually reviewed. Using natural language processing (NLP) was used. Transcript text was split into sentences and annotated as either statement or question. Sentences were split into individual words, they were then compared against task-specific verbal cues associated with general CPR, depth of compressions, rate of compressions, or positive feedback. If a word matched a CPR term, it was annotated with the corresponding label. These annotations separated directive utterances that corrected poor performance from those containing positive feedback for a given CPR component. All annotations were manually reviewed for accuracy.	NLP was successfully used to analyze transcripts of team interactions.<br><br>Coached groups had increases in the total number of unspecified positive, rate directional, depth-positive, and depth-directional utterances per minute (1.34 vs. 0.55, 0.21 vs. 0.04, and 0.63 vs. 0.16, respectively, $p < 0.05$ in all comparisons). Coached groups also had fewer total questions asked (2.84) per minute vs. 3.66 per minute, $p < 0.05$).<br><br>The frequency of depth cues from coaches adapted over time, indicating they adjusted their feedback as CPR progressed.<br><br>In coached teams, the number of depth cues varied depending	Missing non-verbal cues: Tone of voice and body language were not captured.<br><br>Simulation setting: The study was based on simulated resuscitation sessions, which may not reflect real-world conditions.<br><br>Audio quality: A single microphone was used, limiting the quality and accuracy of audio capture, which may have affected transcription.

	non-coached).<br><br>Recorded on video camera, CPR quality data collected from Zoll R Series feedback defibrillator.	For each conversation, 18 data points (18 for each of the 40 teams) rep-<br><br>resenting the percentage of excellent CPR over a minute-long interval<br><br>were provided as outcome measures from the original trial, totaling 720<br><br>team-minutes.	on the pattern of CPR excellence, suggesting a tailored approach to feedback.	
Molu, 2025, Turkey[18]	To compare the efficacy of an artificial intelligence (AI)-based care plan learning strategy with standard training techniques in order to determine how it affects nursing students' learning results in newborn resuscitation.<br><br>70 third-year nursing students<br>Experimental group received care plans based on AI, and the control group received traditional instruction (prepared by researchers). Both groups received 4-week neonatal resuscitation training prior (2h theoretical sessions per week).<br><br>Quasi-experimental randomized study with a pre-test–post-test control group.<br><br>Neonatal Resuscitation Knowledge test	The researcher entered basic commands and information about neonatal resuscitation into ChatGPT and asked the system to write five disease stories containing signs and symptoms that may cause neonatal resuscitation. A free research preview of ChatGPT from Open AI that was last updated on September 2021 was used to produce the outputs. The evaluation process involved a careful review of each case scenario and the corresponding care plan. Students were required todevelop their care plans based on these personalised AI-generated case scenarios. The AI-based care plans allowed students to engage with a variety of neonatal resuscitation challenges, providing a more adaptive and customised learning experience.<br><br>The traditional care plans were static and did not change based on individual student performance.	After analysing the mean scores of the'traditional education' and<br><br>'AI-based education' groups of students, the study found that the<br><br>groups' pre-test scores were similar and that, in the post-test ($p < 0.05$), the AI-based education group outperformed the traditional education group by a significant margin.	
